# Effects of Different Comorbidities on Health-Related Quality of Life among Respiratory Patients in Vietnam

**DOI:** 10.3390/jcm8020214

**Published:** 2019-02-07

**Authors:** Chau Quy Ngo, Phuong Thu Phan, Giap Van Vu, Quyen Le Thi Pham, Long Hoang Nguyen, Giang Thu Vu, Tung Thanh Tran, Huong Lan Thi Nguyen, Bach Xuan Tran, Carl A. Latkin, Cyrus S. H. Ho, Roger C. M. Ho

**Affiliations:** 1 Department of Internal Medicine, Hanoi Medical University, Hanoi 100000, Vietnam; ngoquychaubmh@gmail.com (C.Q.N.); thuphuongdr@gmail.com (P.T.P.); drgiap@hmu.edu.vn (G.V.V.); phamlequyenbmh@gmail.com (Q.L.T.P.); 2 Center of Excellence in Behavioral Medicine, Nguyen Tat Thanh University, Ho Chi Minh City 700000, Vietnam; longnh.ph@gmail.com (L.H.N.); pcmrhcm@nus.edu.sg (R.C.M.H.); 3 Center of Excellence in Evidence-based Medicine, Nguyen Tat Thanh University, Ho Chi Minh City 700000, Vietnam; giang.coentt@gmail.com (G.T.V.); tung.coentt@gmail.com (T.T.T.); 4 Institute for Global Health Innovations, Duy Tan University, Da Nang 550000, Vietnam; huong.ighi@gmail.com; 5 Institute for Preventive Medicine and Public Health, Hanoi Medical University, Hanoi 100000, Vietnam; 6 Johns Hopkins Bloomberg School of Public Health, Baltimore, MD 21205, USA; carl.latkin@jhu.edu; 7 Department of Psychological Medicine, National University Hospital, Singapore 119074, Singapore; cyrushosh@gmail.com; 8 Department of Psychological Medicine, Yong Loo Lin School of Medicine, National University of Singapore, Singapore 119228, Singapore

**Keywords:** respiratory diseases, comorbidity, HRQOL, minimally clinically important difference, Vietnam

## Abstract

Comorbidities are common in respiratory disease patients and have been well-known to impact their quality of life. The objective of this study is to estimate the minimal clinically important difference (MCID) of the health-related quality of life (HRQOL) among respiratory disease patients with different comorbidities in a Vietnamese tertiary hospital. We performed a cross-sectional study from October to November 2016 at the Respiratory Center of Bach Mai Hospital, Hanoi, with a total of 508 participants. Information about socio-economic characteristics, HRQOL and comorbidities of participants was collected. ANOVA was used to identify MCID between patients with and without specific comorbid conditions. Tobit regression was used to explore the associations between comorbidities and the HRQOL. Results showed that the prevalence of cardiovascular comorbidities was 23.8%, followed by musculoskeletal diseases (12.0%), digestive diseases (11.8%), endocrine diseases (10.0%), kidney diseases (5.1%) and ear, nose, and throat diseases (4.5%). Regarding HRQOL, having a problem in pain/discomfort was observed in 61.0% of participants, followed by anxiety/depression (48.2%). Mean EQ-5D index was 0.66 (SD (Standard Deviation) = 0.31). The significant MCID (*p* < 0.05) was found between patients with and without cardiovascular diseases, musculoskeletal diseases, kidney diseases, and endocrine diseases. The multivariate regression model showed that only musculoskeletal diseases were found to be related with the marked decrement of EQ-5D index score (Coef. = −0.13; 95% CI (Confident Interval) = −0.23; −0.02). Suffering at least one chronic illness was correlated to the marked decrease of EQ-5D index score (Coef. = −0.09; 95% CI = −0.17; −0.01). These results underline the importance of appropriate pain management as well as the provision of an interprofessional care approach to patients in order to alleviate the burden of comorbidities to their treatment outcomes and HRQOL.

## 1. Introduction

World Health Organization defines chronic respiratory diseases which are related to impairments of airways and lung, characterized by symptoms of breathing difficulties, repeatedly coughing, and excessive mucous [1]. In chronic respiratory diseases, patient-reported outcomes, particularly the health-related quality of life (HRQOL), are recommended to be evaluated along with clinical and laboratory indicators [2]. The HRQOL measured by standardized instruments can inform impacts of specific health conditions on patients’ lives and well-being [3]. Respiratory diseases such as chronic obstructive pulmonary disease (COPD) and asthma have been well-known to diminish remarkably patients’ HRQOL [4,5,6]. 

Comorbidities such as anxiety disorders, diabetes or cardiovascular diseases have been frequently observed in patients with respiratory diseases [7,8,9]. They have been shown to increase the risk of hospitalization, treatment cost and mortality [10,11,12,13]. Regarding the HRQOL, previous studies suggested that these comorbidities considerably reduced the HRQOL of patients with COPD [4,14,15,16]. However, studies to explore the effects of comorbid conditions on the HRQOL in general respiratory patients regarding different organ systems are limited, requiring further investigations to fill this gap. 

Measuring HRQOL only does not provide clinical differences among distinguished health conditions or treatments in clinical settings. One concept that has received attention in the literature is minimal clinically important difference (MCID). This term refers to the smallest difference (i.e., the effect size) of two health conditions, treatments or interventions that is considered meaningful for patients [17,18]. This concept is crucial by interpreting the meaning of HRQOL outcomes in clinical perspective rather than statistic perspective, especially in the cases that involve multiple comorbidities [18].

In Vietnam, respiratory diseases can be considered a major health issue, being the fourth most common causes of death for Vietnamese people [19]. However, there has been a lack of studies determining MCID of respiratory patients’ HRQOL with different comorbidities. Therefore, the objective of this study is to estimate the MCID of the HRQOL among respiratory disease patients with different comorbidities in a Vietnamese tertiary hospital. The findings of this study would potentially be used to suggest innovative screening/care approaches for patients with respiratory diseases. 

## 2. Experimental Section

### 2.1. Study Design and Sampling

We conducted a cross-sectional study from October to November 2016 at Respiratory Center of Bach Mai Hospital, Hanoi. This Respiratory Center is one of the major facilities that treat respiratory diseases in the North of Vietnam. The center serves a diverse range of patients who are referred by or voluntarily moved from provincial/communal level facilities in other provinces due to the severity of their conditions.

Patients who met the following criteria were selected for participating in the study: (1) 18 years old or above (2) Being treated at the Respiratory Center either an inpatient or outpatient department; (3) Being able to answer the questions from interviewers for 5 to 10 min; (4) Agreeing to participate in the study and give informed consent. A total of 508 patients were recruited to participate in the study. Participants were interviewed face-to-face by trained interviewers. They were asked to give their written informed consents. They were also informed that they could withdraw at any time without any impacts on their current treatment. The protocol of the study was approved by the Institutional Review Board of Vietnam Respiratory Society (Reference code: 06/QD-VNRS).

### 2.2. Measurements and Instruments

A structured questionnaire was utilized to collect information of respondents. Patients agreeing to enroll in the study were invited to a private room to ensure confidentiality and quality of the answers. Each interview lasted 5–10 min. We collected the following information:

#### 2.2.1. Socio-Economic Characteristic

Socio-economic characteristics including gender, education, marital status, employment, living area and age were collected.

#### 2.2.2. Health-Related Quality of Life (HRQOL) and Comorbidities

The Europol-5 dimensions-5 levels (EQ-5D-5L) measure was employed to evaluate patients’ HRQOL at the day of interview regarding Mobility, Self-Care, Usual Activities, Pain/Discomfort, Anxiety/Depression. There are five levels of response in each domain from “no problem” to “extremely problem”; and combining the responses of five domains produces health states with corresponding single index [1]. In addition, the information on the comorbidities of respondents was imported from their medical records.

### 2.3. Statistical Analysis

Stata version 15.0 was used to analyze the data. ANOVA test was employed to compare the HRQOL of patients having or not having specific comorbidities. In this study, comorbidities with 10 patients or more were included. This approach was used in previous studies among Vietnamese and Japanese general populations aiming to assure the reliability of the MCID estimate [20,21]. However, we found that most of comorbidities were presented in less than 10 patients; hence, these comorbidities were classified into six groups including: cardiovascular, digestive, musculoskeletal, kidney, endocrine and ear, nose, and throat diseases. We excluded other comorbidity groups having less than 10 patients such as dermatology or oncology diseases to ensure the quality of analysis [21]. The anchor-based approach was used to measure the MCID since it has been used in previous cross-sectional studies [20,21]. If patients had two comorbid conditions or more, we matched them with other patients with similar conditions but without different targeted comorbidities. For instance, if a patient had both kidney and cardiovascular diseases, we matched s/he with another patient with similar socio-demographic characteristics and kidney diseases only in order to estimate the MCID of the HRQOL due to cardiovascular diseases. Multivariate Tobit regression was employed to identify comorbidities that were associated with HRQOL of respondents. A *p*-value < 0.05 was used as a threshold for detecting statistical significance.

## 3. Results

Of 508 patients, the mean age was 54.6 (SD = 17.5). Most of the respondents were males (56.9%). There were 83.7% married participants. The proportion of patients having under high school education was the highest with 55.1%. Most of patients lived in rural areas (65.8%). The proportion of patients being farmers or unemployed was 34.1% and 34.1%, respectively (Table 1).

HRQOL and commodities of patients are presented in descending order in Table 2. The proportion of patients having cardiovascular diseases was the highest with 23.8%, following by musculoskeletal diseases (12.0%), digestive diseases (11.8%), endocrine diseases (10.0%), kidney diseases (5.1%) and ear, nose, and throat diseases (4.5%). There were 32.7% having one comorbidity, 12.0% and 3.3% having two and three comorbidities or more, respectively. Regarding HRQOL, having the problem in pain/discomfort was observed in the majority of patients at 61.0%, followed by anxiety/depression (48.2%), usual activity (36.2%), mobility (33.1%) and self-care (31.3%). The mean EQ-5D index was 0.66 (SD = 0.31). 

**Figure 1:**
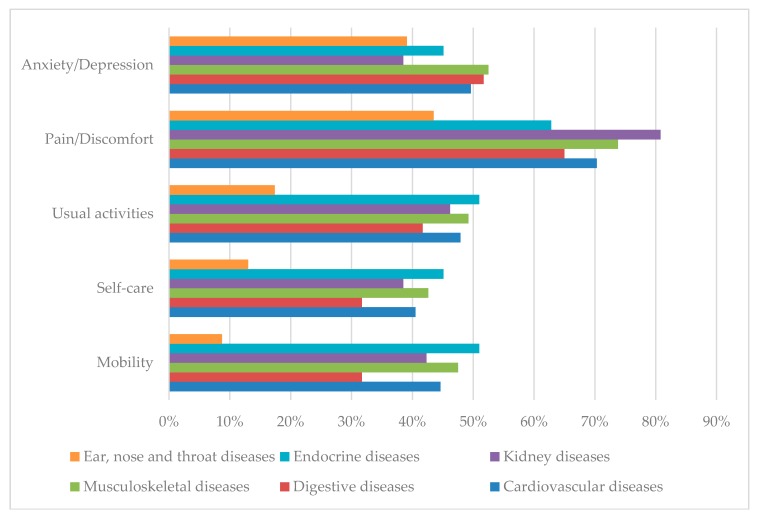
Proportion of patients reporting any problems in the Euroqol-5 dimensions-5 levels (EQ-5D-5L) dimensions by different chronic diseases.

Figure 1 reveals the proportion of patients experiencing any problems on EQ-5D-5L dimensions by different comorbidities. The rates of patients with ear, nose and throat diseases suffering problems in four dimensions (mobility, self-care, usual activities, and pain/discomfort) were the lowest. Respondents having endocrine diseases suffered the most problems in mobility, self-care, and usual activities. 

**Figure 2:**
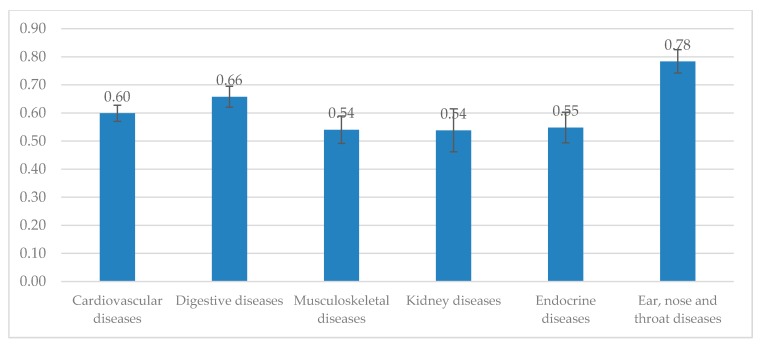
EQ-5D-5L index by chronic conditions.

In Figure 2, the mean EQ-5D index was the lowest among patients with kidney diseases (EQ-5D index = 0.54) and musculoskeletal diseases (EQ-5D index = 0.54). Meanwhile, the highest EQ-5D index was in respondents with ear, nose and throat diseases (EQ-5D index = 0.78) and digestive diseases (EQ-5D index = 0.66).

**Figure 3:**
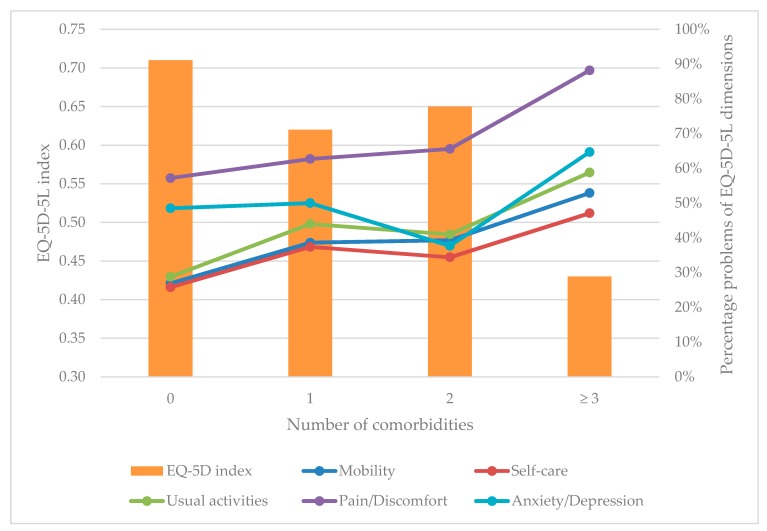
EQ-5D-5L dimensions and index according to the number of comorbidities.

Figure 3 shows that patients not having comorbidities had the mean EQ-5D index = 0.71, followed by those having two comorbidities (EQ-5D index = 0.65) and one comorbidity (EQ-5D index = 0.62). Patients having three comorbidities or more had the lowest EQ-5D index with 0.43. Overall, those having a higher number of comorbidities experienced more problems in all dimensions of EQ-5D-5L instrument. 

Table 3 shows that the MCIDs between patients with and without cardiovascular diseases (diff = −0.09), musculoskeletal diseases (diff = −0.14), kidney diseases (diff = −0.13), endocrine diseases (diff = −0.13), having one (diff = −0.10) and three diseases or more (diff = −0.28) were statistically significant.

Table 4 shows that in univariate Tobit regression, cardiovascular diseases, musculoskeletal diseases, kidney diseases, and endocrine diseases were related to a remarkable decrease in the EQ-5D index. The highest reductions were among patients having musculoskeletal diseases (Coef. = −0.17; 95% CI = −0.28; −0.06) and Kidney diseases (Coef. = −0.17; 95% CI = −0.33; −0.01). However, after adjusting to age, sex, education, marital status, occupation and living location in multivariate model, only musculoskeletal diseases were found to be related with the marked decrement of EQ-5D index score (Coef. = −0.13; 95% CI = −0.23; −0.02). 

In comparison with those not having comorbidity, having at least one disease were associated with the significant decrease of EQ-5D index score (Coef. = −0.09; 95% CI = −0.17; −0.01). Those having multi-comorbidities (at least two comorbidities) reported lower EQ-5D index score compared to their counterpart (Coef. = −0.05; 95% CI = −0.14; 0.05), however, this reduction was not statistically significant.

## 4. Discussion

In this study, our findings highlight a significant clinical reduction in HRQOL among respiratory patients with different comorbidities, particularly musculoskeletal diseases, after controlling socio-demographic characteristics. Results of this study might support clinicians in determining clinical improvement when treating respiratory diseases with different comorbidities.

The current study found that health utility score had a reduction of 0.09 among patients having one comorbidity; and 0.27 among patients with three comorbidities or more. These results are aligned with the previous study in the general Vietnamese population, which a decrement of 0.07 in HRQOL measuring by EQ-5D-5L was observed among people with one morbidity [20]. Our result was also similar to the study among the general Canadian population [22]. Moreover, suffering musculoskeletal diseases was found to have a significant clinical reduction in HRQOL in respiratory disease patients, with a decrement of 0.14. The musculoskeletal dysfunctions might lead to the reduction of patients’ mobility, and the increase of dyspnea and fatigue levels might decrease patients’ HRQOL [23,24,25]. This was consistent with the finding in the general population, which revealed a reduction of 0.15 in EQ-5D utility score [20]. Therefore, an increase of 0.14–0.15 on the EQ-5D-5L index could be considered a clinically important change for the musculoskeletal diseases in different Vietnamese populations. 

Notably, despite significant differences when assessing between-group MCID, effects of cardiovascular, kidney and endocrine diseases on the change of HRQOL were not found in the multivariate regression. The reasons might be due to the fact that these diseases which patients suffered were mild or did not significantly cause pain/discomfort or anxiety/depression compared to musculoskeletal diseases. Further studies for the effects of specific diseases with their severity should be elucidated. 

The findings of this study suggest several implications. First, causes of pain/discomfort-related respiratory diseases should be understood to provide appropriate pain management strategies since pain/discomfort was the most common health problem among these patients. Psychological counseling service should also be provided in order to screen and prevent the occurrence of depression/anxiety due to illness in respiratory disease patients. Second, a standardized and validated measure such as EQ-5D-5L should be integrated into clinical routine, which helps to evaluate the improvement of HRQOL and identify the reasons of decrement to ensure the effectiveness of treatment. Third, as comorbidities were common among patients with respiratory diseases, an interprofessional care approach should be considered in delivering health care services to these patients. Finally, our results could be informed as an integral part of treatment outcomes among patients with respiratory diseases, which could possibly be used in further health economic evaluations. 

To our knowledge, this is one of the first studies investigating the MCID of the EQ-5D-5L in patients with respiratory diseases even though it was previously applied in the Vietnamese general population [20]. In this study, the anchor-based approach was used to measure the between-group MCID of HRQOL among those with and without specific comorbidities. It was argued that this method could be applied in both longitudinal and cross-sectional assessments [26], which was more pervasive in the former design. Our constrained resources would not allow us to conduct repeated measures as recommended. However, some authors claimed that information from our approach could be potentially utilized with suitable interpretations of the finding [20,21]. For example, as discussed above, our results are comparable to the findings in the Vietnamese general population, which had results that were consistent with other longitudinal studies [20].

Several limitations in the current study should be acknowledged. First, we did not include variables about the severity and duration of illnesses, which might have significant associations with the HRQOL [27]. Second, because of the nature of the cross-sectional design, we were unable to conclude the causal relationships between the changes of HRQOL and different comorbidities among respiratory disease patients. Thus, further longitudinal studies should be required to confirm the impacts of comorbidities on the HRQOL of patients with respiratory diseases. Third, our study setting was a tertiary hospital and we employed the convenient sampling method; thus, our results should be used with caution when applying in other settings since this limitation might restrict our generalizability. 

## 5. Conclusions

In conclusion, our study reveals that comorbidities, particularly musculoskeletal diseases, were related to clinically important reductions in the HRQOL of patients. Moreover, pain and discomfort were the most critical health issues among patients with respiratory diseases at the tertiary hospital setting. These results underline the importance of appropriate pain management as well as the provision of an interprofessional care approach to patients in order to alleviate the burden of comorbidities to their treatment and HRQOL.

## Figures and Tables

**Table 1:** Socio-economic characteristics of respondents (*n* = 508).

Characteristics	*n*	%
Gender, Male	289	56.9
Education		
Under high school	280	55.1
High school	127	25.0
Above high school	101	19.9
Marital status		
Married	425	83.7
Single	83	16.3
Employment		
Farmers	173	34.1
Unemployed	173	34.1
Freelancers	73	14.4
Workers in public organizations	43	8.5
Workers in private organizations	28	5.5
Others	18	3.5
Living area		
Urban	174	34.3
Rural	334	65.8
	Mean	SD
Age	54.6	17.5

**Table 2:** Health-related quality of life (HRQOL) and comorbidities among respondents.

Characteristics	*n*	%
Comorbidities		
Cardiovascular diseases	121	23.8
Musculoskeletal diseases	61	12.0
Digestive diseases	60	11.8
Endocrine diseases	51	10.0
Kidney diseases	26	5.1
Ear, nose and throat diseases	23	4.5
EQ-5D-5L dimensions		
Pain/Discomfort	310	61.0
Anxiety/Depression	245	48.2
Having problems with usual activity	184	36.2
Having problems in mobility	168	33.1
Having problems in self-care	159	31.3
Number of comorbidities		
0	264	52.0
1	166	32.7
2	61	12.0
≥3	17	3.3
	Mean	SD
Number of comorbidities	0.7	0.8
EQ-5D index	0.66	0.31

**Table 3:** Between-group minimal clinically important difference (MCID) of EQ-5D index scores for different comorbidities.

Health Issue	*n*	EQ-5D Index Score	
		Diff ^†^	95% CI
**Comorbidities**			
No comorbidities	264	-	
Cardiovascular diseases	121	−0.09 *	−0.15; −0.02
Musculoskeletal diseases	61	−0.14 *	−0.22; −0.06
Digestive diseases	60	−0.01	−0.09; 0.08
Endocrine diseases	51	−0.13 *	−0.22; −0.04
Kidney diseases	26	−0.13 *	−0.26; −0.01
Ear, nose and throat diseases	23	0.12	−0.01; 0.25
**Number of comorbidities**			
0	264	-	
1	166	−0.10 *	−0.17; −0.02
2	61	−0.07	−0.18; 0.05
≥ 3	17	−0.28 *	−0.48; −0.09

^†^ Difference between respiratory patients with and without different chronic conditions. * *p* < 0.05.

**Table 4:** Correlations between EQ-5D-5L index and Comorbidities.

Characteristics	Model 1	Model 2
	Coef (95% CI) ^a^	Coef (95% CI) ^b^
**Comorbidities**		
Cardiovascular diseases	−0.11 (−0.19; −0.02) *	−0.06 (−0.14; 0.03)
Digestive diseases	−0.01 (−0.12; 0.10)	−0.01 (−0.12; 0.10)
Musculoskeletal diseases	−0.17 (−0.28; −0.06) *	−0.13 (−0.23; −0.02) *
Kidney diseases	−0.17 (−0.33; −0.01) *	−0.14 (−0.30; 0.01)
Endocrine diseases	−0.15 (−0.27; −0.03) *	−0.11 (−0.22; 0.01)
Ear, nose and throat diseases	0.15 (−0.03; 0.32)	0.10 (−0.08; 0.27)
**Number of comorbidities**		
0	Ref	Ref
1	−0.12 (−0.19; −0.04) *	−0.09 (−0.17; −0.01) *
2	−0.08 (−0.19; 0.03)	−0.03 (−0.15; 0.08)
3	−0.34 (−0.54; −0.15) *	−0.27 (−0.46; −0.08) *
**Multimorbidity (≥2 diseases)**		
No	Ref	Ref
Yes	−0.09 (−0.19; 0.00)	−0.05 (−0.14; 0.05)

^a^ Crude Coefficient; ^b^ Adjusted to age, sex, education, occupations, marital status, and living location. * *p* < 0.05.
